# Low-Level Laser on Hearing: Is There an Effect?

**DOI:** 10.1155/2013/839256

**Published:** 2013-11-11

**Authors:** Jan Tunér, Lars Hode

**Affiliations:** ^1^Private Dental Clinic, Spjutvagen 11, 772 32 Grängesberg, Sweden; ^2^Swedish Laser Medical Society, P.O. Box 1031, 181 21 Lidingö, Sweden

This is a comment on “*The effect of low-level laser therapy on hearing*.” [1]

The first law of photochemistry, the Grotthuss-Draper law, states that light must be absorbed by a compound in order for a photochemical reaction to take place. Thus, when targeting the cochlea with laser light, it is essential to make sure that a sufficient amount of light reaches the target. Indeed, low-level laser therapy includes secondary effects through systemic mechanisms, but the energy at target is decisive for the biological effect.

The cochlea is located rather deeply in the scull, and to reach it with laser light, different strategies have been used. Some studies have used irradiation via the petrosus bone, but according to the experimental study by Tauber et al. [2], this approach is ineffective. As for transmeatal irradiation, these investigators report great variations in dose at target depending on the position of the probe in the meatus. These authors have therefore used a specially designed laser system for transmeatal irradiation in their pilot study [3], using a wavelength of 632.8 nm or 830 nm, both 20 mW and with the small probe positioned close to the tympanic membrane. After a follow-up period of six months, tinnitus loudness was attenuated in 13 of 35 irradiated patients, while two of 35 patients reported their tinnitus as totally absent. Hearing threshold levels and middle ear function remained unchanged.

The aim of the study by Goodman et al. was not to treat tinnitus, but since studies on the use of LLLT for tinnitus also aim at the cochlea, it is reasonable to compare such studies with the present investigation. The use of low-powered (around 5 mW) red diode lasers for home care has been reasonably successful in some studies but not in all. Investigators using infrared lasers between 300 and 500 mW directly into the meatus report better results [4, 5]. However, most of the studies have failed to perform differential diagnoses between somatosensory (muscular origin, such as TMD) [6–11] and neurological (cochlear dysfunction) backgrounds. Both conditions can be treated with LLLT, but the methods are quite different.

Light in the red spectrum has primarily been used to treat superficial conditions, due to its lower penetration through tissues. Infrared light has therefore been preferred to reach deeper areas, such as the cochlea. Kolárová et al. [12] used CCD camera technique to establish the penetration of 50 mW, 632.8 nm and 21 mW, 675 nm. In the thickest skin sample (19 mm with epidermis + dermis + subcutaneous fat from regio abdominalis) approximately 0.3% of the HeNe and 2.1% of the diode laser light penetrated. So the power of the 7.5 mW red laser light would have reached a level of 0.2 mW after passing through the skin and still not being inside the bone. The penetration of the 532 nm wavelength is three times lower. In the study by Gungor et al. [13], the laser light illuminated the eardrum which is quite transparent and in this case more light reached the cochlea.

Regarding the pulsing used, there is no information about what it is supposed to do and what the chosen pulse repetition rates would do other than reducing the energy per time. In the review on the possible effects of pulsing in LLLT, Hashmi et al. [14] conclude: “It was impossible to draw any meaningful correlation between pulse frequency and pathological condition, due to the wide-ranging and disparate data. As for other pulse parameters, these were in general poorly and inconsistently reported.”

In the study by Goodman et al., the cochlea is reported to be one of the targets of the irradiation. The negative outcome of the study is therefore obvious in that few, if any, photons reached this area or any area of the brain. The combined energy of the two lasers was 15 mW and the energy density was considerably decreased by using a line-generated beam. With an irradiation time of 225 seconds, the total energy was 3.4 J. Given the large area of irradiation, this energy is insufficient even at the surface and at a homeopathic level beneath the bony areas. 

Tinnitus and hearing impairment are common conditions and no conventional effective therapies are available. LLLT appears to have a potential for these conditions, even though the literature is scant and ambiguous. The investigation by Goodman et al. is ambitious and is of high methodological quality. Unfortunately, the chosen treatment parameters are inadequate and we would be happy to see future research by this group but in cooperation with qualified advisors in the field of laser phototherapy.
